# Strong Interaction with Carbon Filler of Polymers Obtained by Pyrene Functionalized Hoveyda-Grubbs 2nd Generation Catalyst

**DOI:** 10.3390/polym11081261

**Published:** 2019-07-30

**Authors:** Annaluisa Mariconda, Anna Agovino, Marco Sirignano, Liberata Guadagno

**Affiliations:** 1 Dipartimento di Scienze (DIS), Università della Basilicata, 85100 Potenza, Italy; 2 Dipartimento di Chimica e Biologia (DCB), Università di Salerno, 84084 Fisciano (SA), Italy; 3 Dipartimento di Ingegneria Industriale (DIIN), Università di Salerno, 84084 Fisciano (SA), Italy

**Keywords:** Hoveyda-Grubbs second-generation catalyst, ROMP, polyolefins functionalization, carbon filler dispersion

## Abstract

Hoveyda-Grubbs 2nd generation catalyst that has the alkylidene functionalized with pyrene (HG2_pyrene_) was synthesized and characterized. This catalyst can be bound to carbonaceous filler (graphite, graphene or carbon nanotubes) by π-stacking interaction, but, since the catalytic site become poorly accessible to the incoming monomer, its activity in the ROMP (Ring Opening Metathesis Polymerization) is reduced. This is due to the fact that the above interaction also occurs with the aryl groups of NHC ligand of the ruthenium, as demonstrated by nuclear magnetic resonance and by fluorescence analysis of a solution of the catalyst with a molecule that simulated the structure of graphene. Very interesting results were obtained using HG2_pyrene_ as a catalyst in the ROMP of 2-norbornene and 1,5-cyclooctadiene. The activity of this catalyst was the same as that obtained with the classical commercial HG2. Obviously, the polymers obtained with catalyst HG2_pyrene_ have a pyrene as a chain end group. This group can give a strong π-stacking interaction with carbonaceous filler, producing a material that is able to promote the dispersion of other materials such as graphite in the polymer matrix.

## 1. Introduction

Polyolefins are widely used materials in many applications such as industrial and food packaging, cling-film and agricultural film, crates, boxes, carrier bags, electrical cable, bottles, pipes, houseware, toys, petrol tanks, carpet fibers, medical appliances, etc., because of their low cost and intrinsic properties of low density, high stiffness, good tensile strength, recyclability, good workability, non-toxicity, and biocompatibility [1]. To enhance these properties and consequently the multi-functionality in advanced applications, it is often necessary to improve the physicochemical and mechanical properties of the polyolefins by introducing fillers into the matrix. In fact, the addition of additives, in particular of nanometric dimensions, has allowed their use for advanced technologies [2,3,4]. Carbon additives such as carbon blacks, carbon nanofiber, carbon nanotubes, and graphene have been largely utilized to improve mechanical properties, thermal stability, flame resistance, and the thermal and electrical conductivities of a pure polyolefin [4,5,6,7,8,9,10,11,12,13,14]. The addition of nanometric filler into polymers enabled important applications in radiation and electromagnetic shielding, antistatic, shrinkage- and corrosion-resistant coatings, light-emitting devices, and batteries [2,3,4,5,6,7,8,9,10,11].

A problem that arises frequently using these fillers is represented by their tendency to form bundles and agglomerates, providing weak interaction within the polymer. Recently, exfoliated graphite or graphene have been tested as promising forms of nanofiller for polyolefins, generating a new class of composite materials with relatively small amounts of nanofiller concentration that can have extensive applications in the aerospace, automotive and construction industries, thanks to the ease of processing and the high strength-to-weight ratio [2,3,4,5,6,7,8,9,10,11]. Graphene has excellent physical-chemical properties: remarkable surface area and optical transmittance [12]. The optical properties of graphene-based nanoparticles demonstrate that they can be used for laser writing. Several intrinsic properties of this nanofiller can be advantageously employed for this last application. Thermal conductivity measurements showed that the incorporation of this allotropic form of carbon in polymeric matrices determines a better heat conduction of the resulting nanocomposites with respect to carbon nanotubes (CNTs) [15]. In addition, it is able to preserve or enhance the thermal stability of the nanocomposites [16,17]. It is also able to confer high thermal and electrical conductivity on insulating hosting polymeric matrices [18]. In light of these considerations, graphene-based nanoparticles can be also used in epoxy-based negative photoresists, both to provide them with functional properties and also as lightweight nanoparticles with metallic behavior embedded into photopolymerizable media to enable better diagnostic methods for examining the shapes of polymerized regions using high-resolution electron-scanning or atomic force microscopies following the optical exposure [19,20,21].

Several articles are available in the literature which show that exfoliated graphite or graphene have been used for the reinforcement of polyolefins obtaining nanocomposites with enhanced properties [14,22,23,24,25,26,27,28,29,30]. The most important aspect in the production of polymeric nanocomposites is the compatibility and dispersion of the nanofiller in the polymer matrix. In fact, a good dispersion, ensuring maximum reinforcement, affects the properties of the entire matrix. The dispersion of graphene in polar polymers has been successfully achieved [5,6,31,32,33], while in the non-polar polyolefins it still represents a significant challenge. In fact, considerable work has been done to obtain homogeneously dispersed nanofiller in polyolefins, without affecting the properties [34,35,36]. Three different techniques may be used: in situ polymerization, solvent blending, and melt processing. In situ polymerization is a widely used technique for the preparation of nanocomposites with insoluble or thermally unstable polymers, which cannot be brought into solution or in the molten state. Generally, an oxidized derivative of graphene (Graphene Oxide, GO) is used as a nanofiller [28,37,38]. Solution blending leads to the mixing of graphene and polymer in a suitable solvent. This is the most used method for preparation of graphene/polymer composites, especially with polymers having high molecular weight. One of the main disadvantages of this method is the use of huge quantities of solvents, which is not environmentally friendly and is hard to completely remove [39]. The melting process is industrially preferred for the production of large-scale nanocomposites, as it is very simple and cost-effective [40]. However, it is less effective in comparison with solution mixing methods, because it is limited to low nanofiller concentrations, since at a higher concentration, the viscosities of the composites are very high, and the workability becomes difficult. Generally, the incorporation of carbon filler into polymeric matrices greatly improves mechanical, electrical, and thermal properties, depending on various factors such as processing techniques, and the type and amount of charged filler. It is important to note that process techniques that are useful for increasing a property could reduce another one, e.g.,: oxygenated functionalities are introduced into the graphite structure by strong oxidizing agents to produce GO, giving rise to a filler that can easily disperse in organic solvents, water, and different matrices. This is a great advantage when using this filler in polymeric matrices to improve their mechanical properties, but compromises the electrical properties of the nanocomposite [41,42]. In this work, a ruthenium-based complex linked to a pyrene unit belonging to the class of second-generation Hoveyda-Grubbs catalysts was synthesized. It binds the carbon filler through π-π stacking interactions and in a non-covalent way. In this manner, the structure of the involved filler is not modified, maintaining all the properties of origin. This should yield a versatile catalytic system that can be linked to any substrate that is able to give such an interaction. It is, therefore, possible to use non-oxidized fillers (graphite, graphene or carbon nanotubes), since oxidation actually reduces conductivity and alters the primitive structure of the carbon filler. However, the most relevant aspect of this work concerns the achievement by the previously mentioned catalytic system, of polymers with a pyrene as a chain terminal. These terminals can provide interaction with the carbon fillers and facilitate the dispersion of the latter in the polymer matrix. The employment of the non-oxidized filler may allow all of the previously mentioned interesting carbonaceous material applications [2,3,4,5,6,7,8,9,10,11,12,13,14,15,16,17,18,19,20,21,22,23].

## 2. Materials and Methods

### 2.1. Materials

All the reactions were carried out in nitrogen atmosphere using standard Schlenk or glovebox techniques. All the reagents used in this study were of reagent grade quality and sourced from Sigma-Aldrich s.r.l. (Milan, Italy). These reagents were used as received. The solvents were dried and distilled before use. Deuterated solvents were degassed under a nitrogen flow and stored over activated 4 Å molecular sieves. The graphite used (trade names Timrex C-Therm 011/012) from Timcal Graphite & Carbon, Bodio, Switzerland; C is a high surface area graphite (trade name Synthetic Graphite 8427) from Asbury Graphite Mills Inc., with a minimum carbon wt % of 99.8 and a surface area of 330 m^2^/g.

### 2.2. Measurements

NMR spectra were recorded on Bruker Avance 400 spectrometer (^1^H, 400 MHz; ^13^C, 100 MHz, Rheinstetten, Germany) operating at 298 K. The samples were prepared by dissolving 10 mg of product in 0.5 mL of deuterated solvent (CDCl_3_ or CD_2_Cl_2_). Tetramethylsilane (TMS) was used as internal chemical shift reference. Spectra are reported as follows: chemical shift (ppm), multiplicity and integration. Multiplicities are abbreviated: singlet (s), doublet (d), triplet (t), quartet (q), multiplet (m) and broad (br). Elemental analysis for C, H, N was recorded on a Thermo-Finnigan flash EA 1112 and were performed according to standard micro-analytical procedures. FT-IR spectra was obtained at a resolution of 2.0 cm^−1^ with a FT-IR (BRUKER Vertex70) spectrometer equipped with deuterated triglycine sulfate (DTGS) detector and a KBr beam splitter, using KBr pellets. The frequency scale was internally calibrated to 0.01 cm^−1^ using a He–Ne laser. 32 Scans were signal averaged to reduce the noise. Thermogravimetric analyses (TGA) were carried out on a TG 209 F1, manufactured by Netzsch Geraetebau, Selb, Germany, at a heating rate of 10 °C min^−1^, under air flow. Fluorescence measurements of the samples were recorded by a Varian Cary Eclipse spectrophotometer. The morphological features were investigated by means of Field Emission Scanning Electron Microscopy (FESEM) analysis. The FESEM characterization was performed with a field emission scanning electron microscope (mod. LEO 1525, Carl ZeissSMTAG, Oberkochen, Germany). 

### 2.3. Preparation of Pyrene-Ru-Based Catalyst

#### 2.3.1. First Step: Synthesis of 4-(1-pyrenyl)-butyryl Chloride (**2**)

**Scheme 1:**
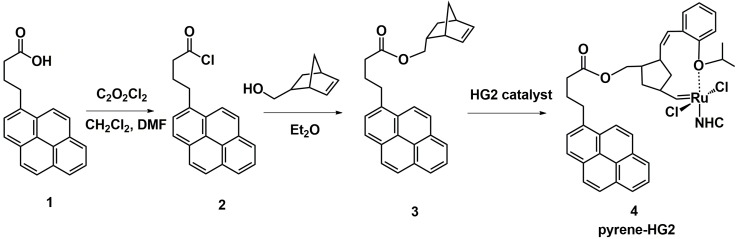
Synthesis of 2nd generation Hoveyda-Grubbs catalyst having the alkylidene functionalized with a pyrene.

4-(1-pyrenyl)-butyryl chloride (**2**), as shown in Scheme 1, was synthesized by treatment of 4-(1-pyrenyl) butyric acid (**1**) (2.0 g, 6.94 mmol) with oxalyl chloride (2.1 mL, 21 mmol) in 225 mL of CH_2_Cl_2_ and two drops of dimethylformamide. The reaction mixture was stirred for 5 h at room temperature. The volume of the solution was reduced under *vacuum* and to the resulting light brown solid was added diethyl ether (22 mL) and hexane (45 mL). The suspension was stirred ca 10 min. The solid was washed with hexane (15 mL). The resultant off-white solid was dried under *vacuum*. Yield: 76%. ^1^H NMR (400 MHz, CDCl_3_, δ): between 8.27 and 7.83 (m, 9H aromatics), 3.43 (t, 2H), 2.98 (t, 2H), 2.33 (m, 2H). ^13^C NMR (100 MHz, CDCl_3_, δ): 174.05 (*C*=O), between 134.68 and 123.16 (aromatic carbons), 46.79 (CH_2_CH_2_*C*H_2_CO), 32.14 (*C*H_2_CH_2_CH_2_CO), 27.06 (CH_2_*C*H_2_CH_2_CO).

#### 2.3.2. Second Step: Synthesis of 4-pyren-1-yl-butyric Acid Bicyclo[2.2.1]hept-5-en-2-yl-methyl Ester (**3**)

Trimethylamine (450 μL, 3.23 mmol) and a crystal of 4-dimethylaminopyridine (DMAP) was added to a stirred solution of 200 μL of 2-norbornene-5-yl-methanol (1.63 mmol) in 50 mL of diethyl ether under nitrogen atmosphere at 0 °C. A solution of 500 mg of 4-(1-pyrenyl)-butyryl chloride (**2**) (1.63 mmol) in 25 mL of diethyl ether was added dropwise. The reaction was carried at room temperature and stirred for 8 h. Deionized water was added at the reaction mixture and the organic layer was dried over anhydrous Na_2_SO_4_, filtered, concentrated, and the residual yellow oil was purified by chromatographic column on silica gel (eluent: hexane/ethyl acetate 5:1). The mixture eluent was removed, and the residue was dried in a vacuum oven. The pure product was isolated in a mixture of *endo*- and *exo*-isomers. Yield: 59%. ^1^H NMR (400 MHz, CDCl_3_, δ): between 8.31 and 7.87 (m, 9H aromatics), between 6.21 and 5.99 (m, 2H), between 4.21 and 3.73 (m, OC*H*_2_, 2H), 3.41 (t, C*H*_2_CO, 2H), between 2.92 and 0.58 (aliphatic protons). ^13^C NMR (100 MHz, CDCl_3_, δ): 173.68 (*C*=O), between 137.82 and 123.52 (sp^2^ carbons), 68.79, 68.11 (*C*H_2_O), between 49.63 and 27.09 (sp^3^ carbons). Elemental analysis of (**3**): found (%): C 85.39; H 6.54; O 8.07. Calc. for C_9_H_13_ON_2_Cl_2_I (%): C 85.25; H 6.64; O 8.11. FT-IR (KBr, cm^−1^): 2964 (s), 2867 (s), 1731 (w), 1631 (s), 1451 (w), 1098 (s), 698 (w).

#### 2.3.3. Third Step: Synthesis of Pyrene-Ru-Catalyst (HG2_pyrene_) (**4**)

The product (**3**) (188 mg, 4.94 × 10^−1^ mmol) and Hoveyda-Grubbs catalyst 2nd generation (HG2) (309 mg, 4.94 × 10^−1^ mmol) at 130 mL of dry CH_2_Cl_2_ were added in a reaction flask. The mixture of reaction was stirred at room temperature for 18 h. After the reaction was completed, the suspension was filtered, and the residue was washed with diethyl ether (3 × 20 mL). The solvent was removed, and the residue was dried in a *vacuum* oven. Yield: 48%. ^1^H NMR (400 MHz, CDCl_3_, δ): 16.57 (s, 1H), 7.95 (br m, 9H, pyrene), 7.49 (t, 1H), 7.07 (s, 4H), 6.85 (m, 3H), 4.90 (m, 1H), 4.17 (s, 4H), 3.92 (br m, 2H), 3.26 (br, 2H), between 2.48 and 1.04 (aliphatic carbons). ^13^C NMR (100 MHz, CD_2_Cl_2_, δ): 211.29 (N*C*N), 173.6_9_ (C=O), between 152.52 and 113.46 (sp^2^ carbons), 75.65 (N*C*H_2_*C*H_2_N), 68.53 (*C*H_2_O), 52.05 (O*C*HCH_3_CH_3_), between 42.06 and 19.67 (sp^3^ carbons), 1.28 (OCH*C*H_3_*C*H_3_). FT-IR (KBr, cm^−1^): 2963 (s), 2917 (s), 2854 (s), 1731 (w), 1631 (s), 1451 (w), 1096 (s), 697 (w).

#### 2.3.4. Functionalization of Graphite with HG2_pyrene_ Catalyst

In an oven-dried Schlenk reaction flask of 100 mL, 200 mg of HG2_pyrene_ (1.63 × 10^−1^ mmol), graphite (1.06 g) and dry CH_2_Cl_2_ (50 mL) were introduced. The mixture was stirred for 2 h at room temperature, then it was filtered and washed with dry CH_2_Cl_2_ (40 mL). The product was recovered and dried in vacuo overnight. Yields 1.09 g.

#### 2.3.5. Evaluation of the Amount of Ru-Based Catalyst Supported on Graphite

The amount of Ru linked to graphite was evaluated by (TGA). The analysis was performed in air flow to allow the Ru to oxidize in the form of ruthenium oxide (RuO_2_). From the residue at 1000 °C, it was possible to calculate the amount of supported catalyst: 6.01 × 10^−8^ mol/mg.

#### 2.3.6. ROMP of the 2-Norbornene and 1,5-Cyclooctadiene

In a typical experiment, a solution of initiator (HG2_pyrene_ or HG2) or suspension of graphite-HG2_pyrene_ (see Table 1) in tetrahydrofuran (1 mL) was mixed with a stirred solution of monomer (2-norbornene or 1,5-cyclooctadiene) in tetrahydrofuran (99 mL). The mixture was kept at room temperature for the times shown in Table 1. The polymerization was stopped with the addition of some drops of ethyl vinyl ether and polymer was coagulated in ethanol. The polymer product was dried under vacuum.

#### 2.3.7. Hydrogenation of Pyrene-Polybutadiene

300 mg of pyrene-polybutadiene (run 5, Table 1) was dissolved in 30 mL of hot-xylene, followed by the addition of 1.50 g of *p*-toluenesulfonylhydrazide. The mixture was stirred at 140 °C for 6 h, filtered, and the hot filtrate poured into methanol (30 mL). The precipitated polymer was recovered by filtration, washed several times with hexane and dried under vacuum to yield the hydrogenated polymer (225 mg).

#### 2.3.8. Interaction of Graphite with Polymers

The polymer was dissolved in chloroform, for 100 mg of polymer were used 20 mL of solvent, the ratio between graphite and polymer was 1:1 by weight. Once the polymer was completely dissolved, the graphite was added, and the solution was stirred for 16 h. The solvent was removed in vacuo.

## 3. Results and Discussion

### Synthesis of Ruthenium-Based Metathesis Catalyst Having the Alkylidene Functionalized with a Pyrene

The modified Hoveyda-Grubbs second-generation ruthenium catalyst (HG2_pyrene_) was obtained using the synthetic strategy reported in Scheme 1, by reacting the suitable pyrene derivative with the classic second-generation Hoveyda-Grubbs catalyst [43,44]. The first synthetic step consists in the reaction of 4-(1-pyrenyl)-butyric acid with oxalyl chloride in order to obtain the 4-(1-pyrenyl)-butyryl chloride (**2**). This compound was esterificated by reaction with 2-norbornen-5-yl-methanol to give 4-pyren-1-yl-butyric acid bicyclo[2.2.1]-hept-5-en-2-yl-methyl ester (**3**). The product (**3**) was characterized by FT-IR, ^1^H and ^13^C NMR analysis and elemental analysis. FT-IR signal at 1731 cm^−1^ attributable to the stretching of the ester group confirms that the reaction occurred. Since, the 5-norbornene-2-methanol used as reagent was in *eso*/*endo* mixture both ^1^H and ^13^C NMR appear rather complex. However, in the ^1^H-NMR at about 6 ppm, the signals of the protons on the double bond of the norbornene are identified, and in the ^13^C NMR it was possible to observe the signals at about 68.79 and 68.11 ppm of the O-*C*H_2_ and the signal at 173.68 ppm attributable to carboxylic carbon. The synthesis of Hoveyda-Grubbs catalyst 2nd generation having the alkylidene functionalized with a pyrene was carried out by metathesis reaction of dichloro-[1,3-bis(2,4,6-trimethylphenyl)-2-imidazolidinyl-idene][(2-isopropoxyphenyl-methylene)-ruthenium(II)] (HG2) with product **3**, obtaining HG2_pyrene_, (see Scheme 1) a stable compound, characterized by NMR analysis. Among the distinctive signals in the ^1^H NMR we should highlight the singlet at 16.57 ppm, which is characteristic of the H-alkylidenic bonded to Ru of the Hoveyda Grubbs catalyst, while in the ^13^C NMR spectrum the signals at 211.29 ppm and at 68.53 ppm of the N*C*N carbene and of the O*C*H_2_, respectively. Furthermore, in the ^1^H-NMR spectrum the signals between 5.99 and 6.21 ppm present in the spectrum of compound **3**, attributed to protons on unsaturated carbons of norbornene ring disappear, confirming the metathesis reaction of norbornene ring with HG2. It is worth noting that in Scheme 1, only the complex with a *cis* double bond between cyclopentane and phenyl ring is shown. Obviously, the ring-opening metathesis of norbornene produces also a *trans* double bond between these rings, as it results from multiplet signal at 6.85 ppm. Thus, through few synthetic steps, it is possible to functionalize the HG2 with the pyrene (HG2_pyrene_). The pyrene group can give a π-stacking interaction with the graphitic plane through solubilization of the catalyst in a suitable solvent, i.e., CH_2_Cl_2_, and then adding the carbonaceous nanofiller at the solution. The mixture is stirred for few hours, then filtered obtaining a catalyst supported on the nanofiller. The occurred formation of the supported catalyst (graphite-HG2_pyrene_) is confirmed by the fact that in the methylene chloride the ruthenium complex is no longer present. The amount of Ru supported on graphite plane was evaluated by thermogravimetric analysis like in previously published papers where Ru-based catalysts were covalently linked to graphene oxide [45,46]. Graphite-HG2_pyrene_ was tested in the ring-opening polymerization of 2-norbornene (Table 1, run 2), performing the same reaction conditions reported for HG2_pyrene_ (Table 1, run 1) and for HG2 (Table 1, run 3). The conversion obtained in run 1 was 84.8%, only slightly lower than obtained by HG2 in run 3, while in run 2 was only 2.63%. A similar decrease in activity was observed by Gomez et al. using a first-generation Grubbs catalyst functionalized with pyrene and making it interact with single-walled carbon nanotubes [47]. It is likely that the π-stacking interaction between the pyrene group and the graphitic plane was very strong and the monomer had difficulty in reaching the catalytic site, thus the activity was drastically reduced. It should be emphasized that, in our catalytic system, the π–π interaction can also occur between the aromatic rings of the NHC nitrogen substituents and the graphitic plane, and this would certainly make the catalytic site difficult to access.

**Figure 1:**
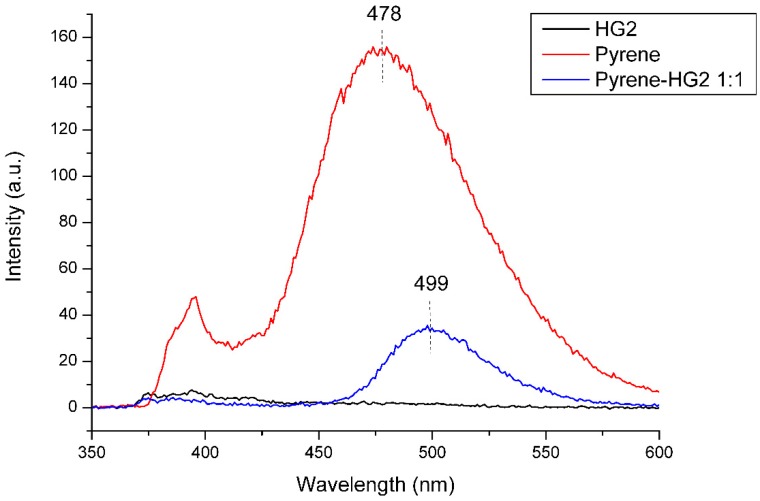
Fluorescence spectra of HG2, pyrene and HG2_pyrene_ molar ratio 1:1 at λ_ex_ 338 nm.

To verify this last hypothesis and the identity of the interaction established between the catalyst and the nanofiller, an investigation by ^1^H NMR and fluorescence analysis with the catalyst HG2 and molecules that simulate the behavior of the nanofiller was carried out. Since the carbon nanofiller is insoluble, we choose the pyrene itself as a molecule which can try to be like the graphitic plane. The pyrene is a fluorophore, in fact, after absorbing photons of a certain wavelength exhibits fluorescence, emitting the absorbed radiation to a different wavelength than the incident radiation. The sample was irradiated at λ_ex_ 338 nm, the excitation frequency of the pyrene. The pyrene, in chloroform solution, shows an intense absorbance at λ 478 nm (red profile in Figure 1) while the catalyst HG2 doesn’t show absorbance (black profile in Figure 1).

The mixture of pyrene/HG2 at molar ratio 1:1 in CHCl_3_ leads to the shift and the decrease of the absorbance of the pyrene at λ 499 nm (blue profile in Figure 1). It is important to note that when an isolated aromatic group form a dimer via the π-stacking interaction, the bathochromic shift is usual. In fact, the excited level of the chromophore isolated, after the dimerization, is divided into a lower and a higher energy level [48]. Furthermore, the interaction can create more non-radiative decay channels and therefore decrease the quantum yield. These two effects evidently indicate that the catalyst establishes a strong interaction with the aromatic network. Probably, this fact makes it difficult for the monomer to reach the catalytic site justifying the low activity of the system [43].

As further confirmation of the interaction π-stacking between the pyrene and the catalyst was obtained by ^1^H NMR analysis. The commercial HG2 catalyst was solubilized in a suitable solvent (CDCl_3_) and introduced into an NMR tube, then pyrene at various concentrations was added (from 1:1 at a 1:4 HG2:pyrene molar ratio).

**Figure 2:**
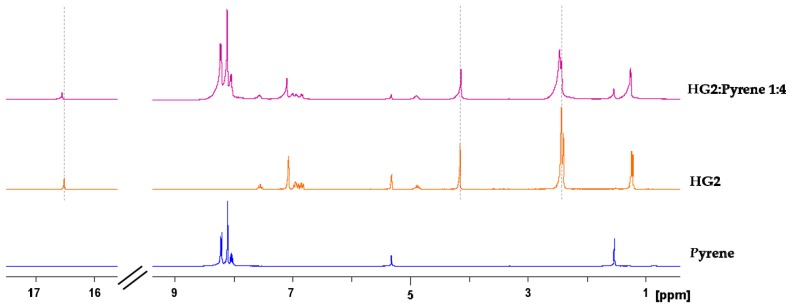
^1^H NMR of pyrene, HG2 and HG2:pyrene at 1:4 molar ratio. TMS scale.

As is shown in Figure 2, almost all the signals undergo a shift that increases with the increase in the concentration of pyrene, e.g., the signal related to the alkylidene proton of HG2 (~16.5 ppm), as well as those of methyls of mesityles (~2.5 ppm) move to lower fields, whereas the signals of methylenes (~4.1 ppm) of the backbone shift to higher fields.

**Figure 3:**
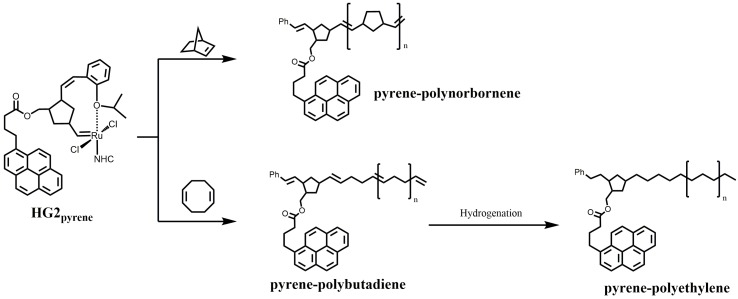
Synthesis of polymers having chain with a pyrene terminal.

Thus, the HG2_pyrene_ system is deactivated when placed together with the graphite, but this does not limit its use as it is. In fact, with this catalytic system two monomers (i.e.,: norbornene and 1,5-cyclooctadiene) were polymerized (run 4 and 5 of Table 1). The polymers obtained have a pyrene as chains end (see Figure 3), so by adding graphite to the polymers dissolved in a suitable solvent, a strong π-stacking interaction between the carbon nanofiller and the polymers was obtained.

Polymerizations were carried out using a monomer/HG2_pyrene_ ratio of 100, in order to obtain short polymer chains, and to maximize the interaction with carbon-filler. The product obtained after polymerizing the 1,5-cyclooctadiene is a polybutadiene with a pyrene terminal. ^1^H NMR analysis shows signals between 8.27 and 7.83, at 5.41 and at 2.03 ppm attributable to hydrogens of pyrene, protons of unsaturated carbons and protons of saturated carbons of the polymer chain, respectively. A fraction of this polymer was hydrogenated to give a pyrene-bonded polyethylene.

The polymers reported in Figure 3 were dissolved in a suitable solvent, then the graphite was added in a 1:1 ratio in mass to allow the formation of the non-covalent interactions. It is interesting to note that, when the graphite is added to the solution, it remains well dispersed even in the absence of agitation. Instead, it tends to deposit on the bottom of the vial either when it is placed alone in the same solvent and when it is mixed with a polymer that does not have the terminal pyrene.

**Figure 4:**
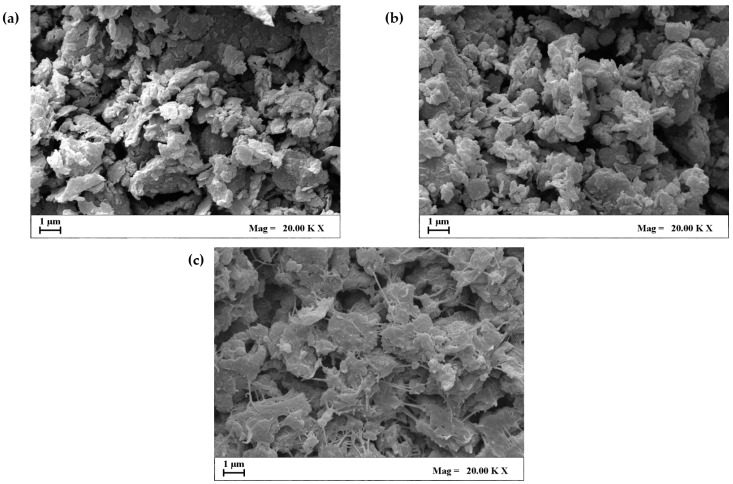
FESEM images of graphite (**a**), of a sample consisting of graphite and of polybutadiene with a terminal pyrene (**b**) and of a sample consisting of polybutadiene (without terminal pyrene) and graphite (**c**).

In order to better understand what was observed, field emission scanning electron microscopy analysis was performed. Figure 4 shows the FESEM images of graphite (a), of a sample consisting of graphite and of polybutadiene with a terminal pyrene (b) and of a sample consisting of polybutadiene (without terminal pyrene) and graphite (c).

From the comparison of the images in Figure 4, it can be seen how in sample b), the polymer is perfectly adhered to the surface of the graphite, which appears to have a very similar aspect to that of the graphite in a). In sample c), there is poor adhesion between the polymer and the graphitic surface, while the interactions between the polymer chains themselves, which then go to form clearly visible separate strands, prevail.

**Figure 5:**
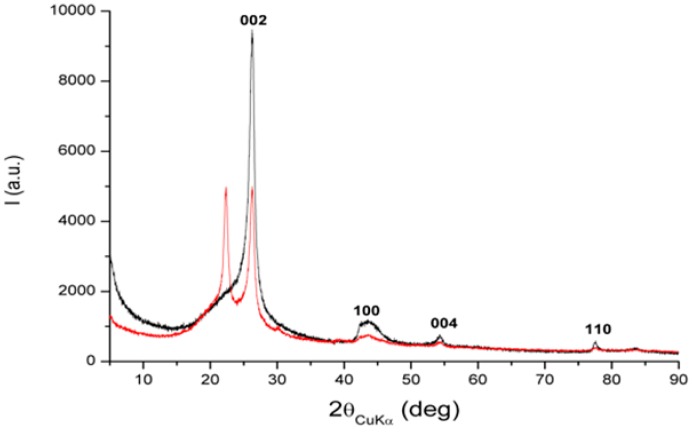
X-ray diffraction patterns (Cu Kα) of graphite+pyrene-polybutadiene (black) and graphite+polybutadiene (red).

The X-ray profile in Figure 5 of graphite shows the characteristic reflection 002 due to the interlayer distance between the graphitic planes, which is d_002_ = 0.34 nm, and a correlation length d_002_ = 6 nm [49]. The in-plane periodicities (d_100_ and d_110_) is well-defined. These parameters do not change by the interaction of the graphite with pyrene-polymer, showing that there is no exfoliation of the graphite. Clearly, the addition of pyrene-polymer determines only a separation between the agglomerated microcrystals. Moreover, it should be noted that the integrity of the graphite layers is preserved even after the non-covalent interactions, in fact, the layer reflexes 100 and 110 remain clear and clearly visible in the diffraction profiles.

The graphite-pyrene-polybutadiene product has been subjected to exhaustive extractions in two different solvents: toluene and xylene. No soluble fraction was recovered for both extractions, indicating that the interaction between the pyrene and the graphitic plane is particularly strong, so it is obviously not necessary to form covalent bonds between the polymer and the carbonaceous nanofiller layer (graphene, carbon nanotubes or exfoliated graphite) to have strong adherence between polymers and these fillers.

## 4. Conclusions

Synthesis of Hoveyda-Grubbs catalyst 2nd generation that has the alkylidene functionalized with a pyrene (HG2_pyrene_) was obtained through few synthetic steps. The products were characterized by FT-IR, ^1^H and ^13^C NMR and calorimetric analysis. The pyrene group can give a strong π-stacking interaction with carbonaceous filler and mixing HG2_pyrene_ with graphite in CH_2_Cl_2_, a catalyst supported on nanofiller (graphite-HG2_pyrene_), was obtained. HG2_pyrene_ and graphite-HG2_pyrene_ were tested in ROMP of 2-norbornene and 1,5-cyclooctadiene. The catalytic activity in the ROMP of system graphite-HG2_pyrene_ was very low because the π-stacking interaction between pyrene group and the graphitic plane was strong and it prevents the coordination of the incoming monomer. The strong interaction between pyrene group and the graphitic plane was demonstrated by NMR and fluorescence analysis.

The catalyst having the alkylidene functionalized with a pyrene (HG2_pyrene_) was, instead, very active in catalysing the ROMP of 2-norbornene and 1,5-cyclooctadiene. The polymers were synthesized have a pyrene as chain end group, which was able to give a strong π-stacking interaction with carbonaceous filler, thus producing a material able to favourite the dispersion of graphite in the polymer matrix.

Therefore, the functionalization by pyrene of the 2nd generation Hoveyda-Grubbs catalyst yields a system capable of producing any polymer obtainable by ROMP with a terminal which makes them capable of binding to carbon fillers, thereby favouring their compatibility.

## Figures and Tables

**Table 1:** Ring Opening Metathesis Polymerization of the 2-norbornene and 1,5-cyclooctadiene.

Run	Initiator	Monomer	Initiator (mol)	Monomer (mol)	Time (h)	Yield (g)	Conversion (%)
1 *^a^*	HG2_pyrene_	NBE	3.84 × 10^−6^	1.13 × 10^−2^	0.029	0.903	84.8
2 *^a^*	Graphite-HG2_pyrene_	NBE	2.10 × 10^−6^	6.85 × 10^−3^	1.5	0.017	2.63
3 *^a^*	HG2	NBE	3.72 × 10^−6^	1.11 × 10^−2^	0.016	0.958	91.7
4 *^b^*	HG2_pyrene_	NBE	7.70 × 10^−5^	6.79 × 10^−3^	0.015	0.637	100
5 *^b^*	HG2_pyrene_	COD	1.27 × 10^−4^	1.22 × 10^−2^	0.010	1.318	100
6 *^b^*	HG2	NBE	4.62 × 10^−5^	4.64 × 10^−3^	0.011	0.435	100
7 *^b^*	HG2	COD	5.06 × 10^−5^	5.08 × 10^−3^	0.030	0.549	100

Conditions: Polymerizations were performed in 100 mL of tetrahydrofuran (THF) at room temperature; NBE 2-norbornene; COD 1,5-cyclooctadiene; *^a^* monomer/initiator ≈ 3000/1; *^b^* monomer/initiator ≈ 100/1.
